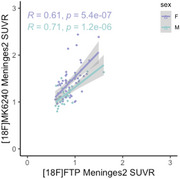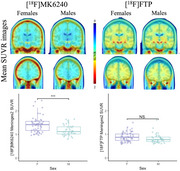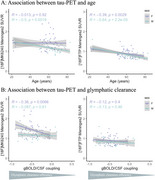# Impact of sex, age and glymphatic clearance on meningeal tau PET signal

**DOI:** 10.1002/alz70856_099912

**Published:** 2025-12-24

**Authors:** Cécile Tissot, Feng Han, Ji Yeon Lee, Hsin‐Yeh Tsai, Nesrine Rahmouni, Joseph Therriault, Stijn Servaes, Jenna Stevenson, Firoza Z Lussier, Brian A. Gordon, Belen Pascual, Val J Lowe, David N. Soleimani‐Meigooni, Hwamee Oh, William E Klunk, Pedro Rosa‐Neto, William J. Jagust, Tharick A Pascoal, Suzanne L. Baker

**Affiliations:** ^1^ Lawrence Berkeley National Laboratory, Berkeley, CA, USA; ^2^ University of California, Berkeley, Berkeley, CA, USA; ^3^ McGill University, Montreal, QC, Canada; ^4^ University of Pittsburgh, Pittsburgh, PA, USA; ^5^ Washington University in St. Louis, School of Medicine, St. Louis, MO, USA; ^6^ Houston Methodist Research Institute, Houston, TX, USA; ^7^ Mayo Clinic, Rochester, MN, USA; ^8^ University of California, San Francisco, San Francisco, CA, USA; ^9^ Brown University, Providence, RI, USA

## Abstract

**Background:**

Variability in meningeal signal has been observed with various tau‐PET tracers. Differences have been observed in sex and age. Furthermore, the potential role of glymphatic clearance in this variability remains unclear. Here we compare meningeal signal of [^18^F]MK6240 and [^18^F]FTP tau‐PET tracers in cognitively unimpaired (CU) amyloid‐β‐ (Aβ‐) individuals, in relation to age, sex and glymphatic clearance.

**Methods:**

92 CU Aβ‐ (7 CU young [18‐35yo] and 85 CU old) underwent [^18^F]MK6240 and [^18^F]FTP tau‐PET scans, structural MRI, and resting‐state functional MRI (rsfMRI). In native space, we defined nine distinct levels representing meningeal regions, organized from those closest to the cortex (1) to those furthest away (9). Glymphatic clearance, measured as gBOLD/CSF coupling, was calculated for each participant; a lower value means a more efficient glymphatic clearance. Associations between meningeal tracer signal and factors such as sex, age, and glymphatic clearance were analyzed using t‐tests and linear regression models.

**Results:**

All 9 meningeal regions were tested and rendered similar results; we will be showing those for meninges level 2. [^18^F]MK6240 and [^18^F]FTP meningeal signal significantly correlated with each other in both sexes (Figure 1). We observed higher [^18^F]MK6240 meningeal signal in females compared to males while no difference was found for [^18^F]FTP signal (Figure 2). In females, [^18^F]MK6240 meningeal signal did not show a significant association with age, whereas in males, a significant relationship was observed. Both males and females exhibited lower [^18^F]FTP meningeal signal with increasing age (Figure 3A). Additionally, glymphatic clearance was significantly associated with age for females with [^18^F]MK6240. No significant associations were found in males with [^18^F]MK6240 or in either sex for [^18^F]FTP (Figure 3B).

**Conclusion:**

Our findings demonstrate that meningeal signal of [^18^F]MK6240 is significantly associated with glymphatic clearance in females, while in males, it is associated with age. In contrast, [^18^F]FTP signal is primarily influenced by age in both sexes. These results highlight the distinct relationship between [^18^F]MK6240 signal and glymphatic function in females, while age appears to play a significant role in both males and females for other tracer signal patterns.